# Building blocks for social accountability: a conceptual framework to guide medical schools

**DOI:** 10.1186/s12909-016-0741-y

**Published:** 2016-08-26

**Authors:** Robyn Preston, Sarah Larkins, Judy Taylor, Jenni Judd

**Affiliations:** 1 College of Medicine and Dentistry, Division of Tropical Health and Medicine, James Cook University, 1 James Cook Drive, Townsville, QLD 4811 Australia; 2 Division of Tropical Health and Medicine, James Cook University, Townsville, Australia; 3 Anton Breinl Research Centre for Health Systems Strengthening, James Cook University, Townsville, Australia; 4 Australian Institute of Tropical Health and Medicine, James Cook University, Townsville, Australia

**Keywords:** Social accountability, Medical schools, Medical education, Conceptual framework, Australia, The Philippines

## Abstract

**Background:**

This paper presents a conceptual framework developed from empirical evidence, to guide medical schools aspiring towards greater social accountability.

**Methods:**

Using a multiple case study approach, seventy-five staff, students, health sector representatives and community members, associated with four medical schools, participated in semi-structured interviews. Two schools were in Australia and two were in the Philippines. These schools were selected because they were aspiring to be socially accountable. Data was collected through on-site visits, field notes and a documentary review. Abductive analysis involved both deductive and inductive iterative theming of the data both within and across cases.

**Results:**

The conceptual framework for socially accountable medical education was built from analyzing the internal and external factors influencing the selected medical schools. These factors became the building blocks that might be necessary to assist movement to social accountability. The strongest factor was the demands of the local workforce situation leading to innovative educational programs established with or without government support. The values and professional experiences of leaders, staff and health sector representatives, influenced whether the organizational culture of a school was conducive to social accountability. The wider institutional environment and policies of their universities affected this culture and the resourcing of programs. Membership of a coalition of socially accountable medical schools created a community of learning and legitimized local practice. Communities may not have recognized their own importance but they were fundamental for socially accountable practices. The bedrock of social accountability, that is, the foundation for all building blocks, is shared values and aspirations congruent with social accountability. These values and aspirations are both a philosophical understanding for innovation and a practical application at the health systems and education levels.

**Conclusions:**

While many of these building blocks are similar to those conceptualized in social accountability theory, this conceptual framework is informed by what happens in practice - empirical evidence rather than prescriptions. Consequently it is valuable in that it puts some theoretical thinking around everyday practice in specific contexts; addressing a gap in the medical education literature. The building blocks framework includes guidelines for social accountable practice that can be applied at policy, school and individual levels.

**Electronic supplementary material:**

The online version of this article (doi:10.1186/s12909-016-0741-y) contains supplementary material, which is available to authorized users.

## Background

Medical schools aspiring towards social accountability deem themselves responsible for meeting the health needs of the communities that they serve^1^ [1]. They orientate their education, research and service to the health needs of the population [2]. From the 1980s to 2000s Dr Charles Boelen and other colleagues at the World Health Organization (WHO) fostered the theory of socially accountable medical education and socially accountable medical schools [3]. These authors then developed the conceptualization, production and utilization grid, the values of social accountability (relevance, equity, cost-effectiveness and quality) and the Towards Unity for Health partnership pentagram [1–10]. From the 2000s to 2010s there was re-interest in measuring and evaluating socially accountable medical schools; including a number of initiatives at individual schools and collectively. These encompassed the Lancet’s Independent Commission on Health Professional Education for the 21st Century [11] and the call for socially accountable medical education to be aligned with accreditation systems.

Despite the advancement of conceptual frameworks, many medical schools aspiring to social accountability had developed independently from the theoretical concepts of socially accountable medical education. There are gaps in the literature around the key factors, both externally and internally, influencing medical schools advancing towards social accountability and the way in which they operate. While the theory of social accountability identifies key principles, how these play out in different schools is the topic of this paper. We present a conceptual framework, derived from empirical research from practice, describing the building blocks for socially accountable medical education.

## Methods

A social justice perspective, drawing upon constructivism and critical theory, was utilized throughout this research. This perspective is linked with the phenomena of social accountability and the epistemological position of the researchers [12, 13]. The voices of all participants had to be heard and given equal weight in contributing to new knowledge. This was important as this research included cases from more financially and less financially resourced countries. Recent literature in medical education has criticized global medical school partnerships as post-colonialist or neo-imperialist enterprises, with imbalances in power relationships and benefits [14, 15]. Furthermore, the powerful profession of ‘the medical doctor’ was investigated. Issues of power and professionalism are played out between and within health professionals [16]. One way in which socially accountable medical education has been defined and implemented is in the context of medical dominance and the changing role of ‘the doctor’ as a professional and ‘leader’ in the health world [16–21]. The authors attempted to actively acknowledge and confront that research can reproduce or enforce existing power structures. Researchers aimed to be self-reflective and self-critical of the values and interest that underlie social theory [22] and explicitly declare their own assumptions [23].

This project utilized multiple case studies [13, 24–26] of four medical schools in Australia and in the Philippines.^2^ Cases were sampled on the basis of maximizing the likelihood of demonstrating a range of contextual influences [13] and membership of the Training for Health Equity Network (THEnet), a collaboration formed in 2008 of 11 health professional schools aspiring towards social accountability^3^ [27–32]. Seventy-five purposively sampled faculty, students, health professionals and community members were interviewed (Table 1) on their understanding of social accountability and what contextual factors had influenced their schools. From a review of the literature these were hypothesized to be:

**Table 1:** Individual Participants [3]

Type	Description	Participants				
		Case 1 FUSOM	Case 2 JCUSMD	Case 3 ADZUSOM	Case 4 UPMSHS	Total
Staff/Faculty	“Champions at schools.” Any staff members who know about social accountability and/ or the history of the school, including leaders and former leaders.	11	11	7 (including 2 former students)	9 (including 2 former students)	38
Students	Student at any year level who are interested in social accountability. In Australia, students who were involved in the rural and/or international health student groups.	4	4	5	4	17
Health Sector	A person holding a position involved in policy, medical education at a state, regional or provincial level, who has had involvement in the school. A preceptor or someone from a hospital or the health department, who has been involved with the school. At two schools (UPMSHS and ADZU) this included Graduates of the school who may also have a teaching or preceptor role.	2 (including 1 preceptor)	2 (including 1 joint JCUSOM appointment with Department of Health)	6 (all had teaching roles and all were former students)	4 (including 3 former students; all involved in teaching students)	14
Community	“Champions at schools.” Any community person involved in the school in teaching or research or at rural placement sites.	1	1	2	2	6
Total		18	18	20	19	75

- Profile of the local health workforce;
- Partnerships with the local, state and national health system; and
- Partnerships between the medical school and its ‘community’.

(Additional file 1 outlines examples of the interview guides). Site visits were undertaken by the principal researcher (RP) to all four schools, including a rural area at each location. Documents analysed included peer reviewed papers, meeting reports, websites and policies [33].

Data analysis was abductive and involved deductive and inductive iterative thematic analysis of qualitative data [34], both within case and across cases [25, 35]. All interviews were transcribed verbatim and any changes identified by participants during verification were incorporated into the transcripts. Field notes, and notes from documentary analysis were transcribed and NVivo 10 was used to store, organize and analyze data [36]. Within case analysis was undertaken by abductive coding; the combination of a predefined deductive coding framework (set by the questions and the three contextual issues obtained from the literature review) and open coding that recorded emerging ideas [3, 37]. Themes were developed and finalized through the constant comparison method, drawing out amalgamated ideas from the codes. Initial similar themes across cases were developed from data immersion. Themes were refined and connections between themes mapped using site-order descriptive matrixes for each of these questions [38]. Coder triangulation was undertaken and negative instances that challenged initial assumptions and interesting outliers were identified to ensure that conclusions were drawn from the data [39]. Themes that emerged consistently across all data were categorized as core themes. Concepts were then further developed in relation to the research questions and from the literature. There were consistent themes across all cases, regardless of the country or other factors. This paper reports on the cross-case analysis.

## Results

**Local and national workforce issues** and **health needs** were important external contextual factors that influenced all four socially accountable medical schools:

*So workforce was the reason the government funded it* [the medical school] *so absolutely if you move back from government funding* [it] *was workforce, workforce, workforce…* (FU1_staff).

Workforce needs were defined as shortages of doctors in underserved areas and the mal-distribution between urban and rural areas. All institutions had geographic proximity to a defined region of workforce need, or sent their students there for a lengthy period, often coexisting with populations with the poorest health indicators or greatest health needs. Programs and schools were established to meet these workforce and health care needs and there were some innovations in both the Philippines and in Australia. The workforce and health system needs influenced how students were trained and the desired outcomes of their training. Schools produced graduates who would be well-equipped to meet the workforce and health system needs. Placements ensured that graduates were personally and professionally equipped to work in areas of workforce need.

An important contextual influence came from the **communities** the medical schools served. These differed:

- *At the population level*: Associated with the influence of workforce, at some schools the population of communities and their different health needs had influenced the establishment and orientation of the schools;
- *As communities of place and interest with a formal or informal community engagement policy*: Some schools had formal community engagement policies and informal ways to work with communities of place and interest; sometimes informed by the wider university policy; and,
- *As students becoming part of the communities*: Students undertake placements in underserved communities and are integrated into community life:
  “That’s why you are sent to communities that do not have water, that do not have electricity, to see that not everyone is the same. So that opens our eyes. This is the social situation of our country, not everyone is privileged, not everyone can go to school, and not everyone has the chance to eat three square meals a day. This is our situation in our region and you’re here, you’re privileged to study medicine, it’s for you to help these people” (ADZU2_student).

**Government policy** was an influence in the Australian case studies because workforce need was a political driver. A number of Australian government policies and programs were designed to address rural workforce issues such as rural placements [40–43]:

*In fact you could say that the Commonwealth* [government] *now has* [become], *to some extent, our main champion because without them we would not [have] the money and the policy wouldn’t have developed the way it has and so on* (FU8_Health).

In the Philippines, due to the prevailing culture of export driven medical and health professional education, government policy and programs were not highlighted as influencing socially accountable medical schools.

**Local politics** and **local politicians** were a powerful contextual issue that influenced social accountability at all four medical schools. While the local government sector had more influence in the Philippines, in both countries participants saw the local or first level of government as being part of the community. In the Philippines the health system is devolved with local officials and local politics influencing the budget and this could either impede or enhance student selection, placements, and graduate job placements:

*We need to have a strong partnership or bond with the Local Government Unit (LGU) because it is the LGU that identifies which health workforce they need, so we train the students to become the health workers that they need.* (UPMSHS16_staff).

In Australia, while the local government did not have as much influence over the health system, support was provided through other initiatives that have involved local councils and medical education such as the provision of housing and social support on placements and in some cases, local councils owning primary health care services.

The values and missions of affiliated **universities,** when they were aligned with social accountability, were an important contextual influence. Many of the staff’s own beliefs, including social justice and a religious association, were consistent with overall values. In addition, some schools were part of universities that had a wider institutional culture that encouraged going against the norm to develop innovative programs. For example, difference and freedom from tradition gave FUSOM an opportunity to focus on *“innovation”* or being able to have *“… a different approach to the way they did business [so there] was a fair degree of risk taking on their behalf, innovation, opportunity, that perhaps came with being a new kid on the block”* (FU8_health).

Financial, human and infrastructural **resources** were also a contextual influence in two ways. In some contexts lack of resources has spurred innovation [30, 32]. While both the Filipino schools had received international support at establishment and had continued partnerships with schools in more resourced countries; a lack of resources also promoted self-reliance and a culture of independence and empowerment. There was also an awareness that locals were better equipped to develop the schools; and a creative ability to “*… make do with what we have*” (UPMSHS5_staff). In Australia, government funding was essential for socially accountable programs, such as rural placements, to be established and sustained. Indeed these programs were accurately deemed more expensive than conventional medical education programs due to decentralization to rural areas.

**Leaders** and **individual “champions”** of social accountability influenced the organizational culture of the schools and the type of people attracted to working in the schools and drove innovation. Staff and students were drawn to working with leaders and at institutions that had these values. These leaders’ values, medical experience in the region and personal and professional connections established, re-orientated or sustained the school. Champions from across the health, education and political sectors lobbied for the establishment of new schools or programs:

*…they’re not just people who look at the problem in front of their nose, they take a step back and think. That sort of controlled rage really, is a drive to change things. Because it takes a lot of effort to change the way things are done, so when I say controlled rage I suppose you know the fire in the belly* (JCU12_staff).

All schools were inaugural members of the **Training for Health Equity Network (THEnet)** and therefore had articulated a common understanding of social accountability [28–32]. In general, THEnet legitimized what schools were doing; membership and interaction with this group clarified existing practices and connected the schools with a global movement. All schools had an understanding of resisting traditional models of medical education. Belonging to this group also helped members interpret the high level WHO policies or directives into practical guidelines confirming existing practice. Through THEnet leaders and staff were able to represent their schools on the WHO and other global forums and groups interested in social accountability. Leaders also developed alliances in medical education that assisted with their own professional development.

All schools had an understanding of **resisting traditional models of medical education**. An underlying assumption that was not fully explored by the participants was the idea of being different and defending this difference. This was articulated at JCUSOM as the health and community needs of the geographical north being different. At FUSOM participants discussed the difference and innovation of the university and school. Respondents knew that ADZU SOM had always been against the mainstream medical education. At UPMSHS participants noted they were doing what they could with little resources. The schools naturally challenge orthodoxies, and are seen as alternative models for health professional education [40] but have also faced “institutional isolation and skepticism from more traditional medical schools” [28] (p.340). There was a shared understanding that while the schools may be critiqued for persisting against opposition, subverting dominant paradigms or doing that which was thought impossible by more traditional schools, there was also a defiant confidence that resistance to current models of medical education is ‘the right thing to do’ [27, 44].

## Discussion

### Conceptual framework: building blocks for socially accountable medical schools

**Fig. 1:**
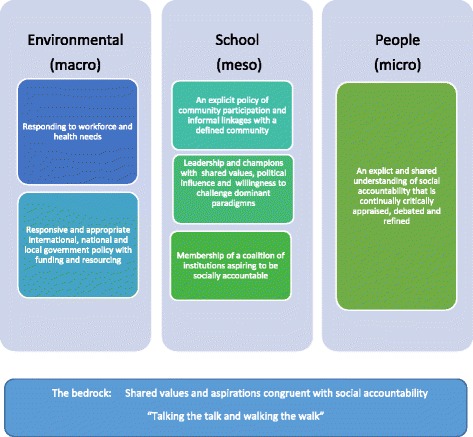
Building Blocks for Socially Accountable Medical Schools

The building blocks conceptual framework (Fig. 1) was created by analyzing the internal and external factors influencing the selected medical schools and demonstrates what ‘building blocks’ or conditions might be necessary to assist medical schools moving towards social accountability. These building blocks are classified as environmental (macro), school (meso) or people (micro) factors. The ‘bedrock’ of social accountability applies at all levels: shared values congruent with social accountability. While many of these building blocks are similar to those conceptualized in social accountability theory, this conceptual framework is informed by what happens in practice - empirical evidence rather than prescriptions. Consequently it is valuable in that it puts some conceptual thinking around everyday practice. These prescriptions are based on data from the four cases and cannot hope to cover all the nuances of practice in widely varying contexts.

## Environmental factors

Environmental factors or macro factors are those outside the medical school. They include the local, regional or national workforce and health needs as well international, national and local government policies and resourcing.

### Responding to workforce and health needs

Workforce was a major influence of schools aspiring to be socially accountable as the main focus of socially accountable medical schools should be meeting workforce needs and understanding the health issues of their region. This finding concurs with the literature on socially accountable medical schools [1, 29, 45]. Schools in areas of workforce need have a more holistic or “whole of school” approach to social accountability as the health system needs are very apparent and hard to ignore.

#### What this means in practice

To understand workforce issues the curriculum of the school should include an analysis of the local workforce situation as well as key health issues. Leaders and staff need to have professional experiences and connections in areas of health and workforce need.

### Responsive and appropriate international, national and local government policy with funding and resourcing

The second building block for socially accountable medical education is the partnership that schools form with government, the health sector, and health care policy makers in health professional education. Governments play different roles depending on the country but the governmental and political context is always important. In Canada, socially accountable medical schools have been mandated in Health Canada policy since 2001 [45, 46]. In more financially resourced countries, such as Canada or Australia, socially accountable programs could not persist without government funding.

In contrast, government policies that have restricted social accountability in the Philippines were overcome due to other factors such as strong leadership and people within innovative schools. The differences in political organization, or between a more centralized and decentralized government, might explain some of the differences in Australia and the Philippines. The decentralized Filipino health system may mean that local initiatives are more influential than government policy.

#### What this means in practice

Medical schools and their leaders need to lobby for medical education and health policies that support the principles of social accountability. For example, for government policies and funding programs that support medical schools in regional and rural settings and in areas where there is workforce need. Medical schools can also adapt and promote government workforce policy or aspects that are socially accountable (for example rural placements). The need for these policies should be promoted and explained in terms of graduate outcomes and long-term impact on health needs of communities. Medical schools should also advocate for a balance of adequate resources with room for innovation and adaptation to the local contexts. Accreditation can motivate medical schools towards social accountability. Medical schools should call for national accreditation systems to include assessment of social accountability. Indeed progress has started on moving the World Federation for Medical Education accreditation standards in this direction [47].

## School factors

School or meso factors include those factors within medical schools including leaders, people (staff/faculty/students) and policies.

### An explicit policy of community participation and informal linkages with a defined community

Community placements, and connections between underserved or rural communities and their health needs, enable students to experience the realities of professional and community life [44, 48–50]. The types of connections between schools and communities vary again depending on the context. The idea of service and links with the community were stronger in the Philippines where there was a sense of personal responsibility. Participants in the Philippines saw the students on placement as ‘helping’ with health and community services. In Australia however the imperative for workforce is more political or organizational and government resources are assigned to this endeavor through programs such as the Rural Clinical Teaching Program [51].

While all schools were influenced by communities, the community may not have appreciated or fully understood the importance of this influence. In addition, the connections may be limited and there might be a lack of authentic engagement and/or partnership. In a critical review Hunt et al. noted that university-community engagement was mainly frequently understood as outreach or service and no articles discussed mutually beneficial or collaborative partnerships with communities [52].

Student placements are one of the major ways through which links are made between rural or underserved populations and the medical school [53]. Community placements were also responsible for changing or confirming student attitudes and aspirations. Long term community-based medical education that develop significant relationships enhances student learning [54].

#### What this means in practice

In order to work effectively with their community a school needs to have a well-defined understanding of the communities, or the defined population that they serve, and an explicit policy about the types of community participation that are appropriate. There will be a need for engaging, partnering or connecting with the community to be undertaken in diverse ways, for example for community to be involved in strategic planning. The community participation strategy of the school needs to be well resourced, achievable and assessed or evaluated.

Generally, schools should partner with non-government or community-based or civil society organizations that already work in communities of need to provide teaching, research, service or health projects. To enable successful partnerships, communities need to be made more aware of their power and influence and community organizations can help facilitate this process.

### Leadership and champions with shared values and political influence and willingness to challenge dominant paradigms

Strong leadership and champions can transform medical education [30, 32, 55] and this is a building block of social accountability. In a study of Deans as “spiritual leaders”, Evans [56] states that leaders should “personify and embody” the values of medicine as a profession and a vocation and “they must remind us of those values and inspire us to embrace them and be guided by them” (p. 655). In promoting social accountability leaders must have not only lived experience as a health professional but “have an iron will to succeed [as] personal experience and integrity are important in the difficult debates that will ensue” [28] (p.10). Institutional or policy barriers to change, including curriculum reform, at medical schools are likely [57–60]. Strong leaders will continue to challenge the dominant paradigms of medical education when they are not supportive of social accountability [61].

#### What this means in practice

In practice, leadership means three things: inspiring socially accountable values; institutionalizing them; and creating a shared understanding of social accountability at the individual, organizational, and community level. The values, medical experience in the region and personal and professional connections of leaders help establish, reorientate or sustain schools. Part of the role of the leader is to institutionalize values and support and to model the culture of social accountability. This is a sustainability step enabling these Schools to embed these values within their culture so that they are not lost when a leader moves on. There needs to be explicit acknowledgment of the type of leadership schools aspiring to be social accountable desire to institutionalize the qualities of leaders. Mentoring aspiring leaders can also ensure socially accountable leadership.

### Membership of a coalition of institutions aspiring to be socially accountable

The role of a network or coalition as a building block to support social accountability is important. The Training for Health Equity Network is a community of practice [40, 62, 63]:

“Communities of practice are groups of people who share a concern, a set of problems, or a passion about a topic, and who deepen their knowledge and expertise in this area by interacting on an ongoing basis” [63] (p. 4).

This community of practice collaborates to solve problems, shares knowledge, creates tools, and has a body of common knowledge, personal relationships, and sense of identity [63]. The influence of THEnet, and other groups of medical schools is that it “can drive change from within” [40] (p. 4) The schools were brought together due to their common goals and challenges and work collaboratively.

#### What this means in practice

Medical schools should join or create a coalition of institutions aspiring to be socially accountable. Coalitions can help foster the professional development of leaders into international medical education leadership roles. Connection with a coalition helps legitimize and develop social accountability, particularly when schools have alternative models of medical education. Other staff and students can be part of a community of practice and receive peer support from colleagues at other schools. A community of practice can help translate or interpret global policies and directives to the local level. These groups can provide opportunities for individual schools to highlight their work. These coalitions should continue to engage schools in the wider social accountability movements such as the WHO and Global Consensus on Social Accountability (GCSA).

## People factors

People or micro factors are the values of people who make up medical schools and their wider institution including staff, students, leaders, health professionals and students. This building block is fully explored in Preston et al. [3].

### An explicit understanding of social accountability

As explored by Preston et al. [3] the difference in perceptions of social accountability indicates that the term may not be universally understood and appreciated [64–70]. Furthermore, the term has danger of losing meaning if schools do not continue to critically appraise and debate the term and what it means in their own unique context.

#### What this means in practice

All stakeholders need to be involved in debating and developing and operationalizing a shared understanding of social accountability to ensure it is socio-culturally appropriate to the context of the school [3]. The school community may argue that the term “social accountability” does not fully capture their mission and practice; particularly in non-English speaking settings. A local term or phase may be developed that captures the nuances and essence of the school culture. Staff students, health sector and community members could be asked “*if our school was socially accountable what would it look like?*” [3] In addition, as discussed by Ritz et al. schools should adopt a “critically reflexive social accountability” [69], (p. 155) that questions underlying assumptions and discourses. Dissenting views and debate should be encouraged as part of this process [3].

The personal values and beliefs of staff, leaders and student influence the organizational culture of the school. People and their values can help drive organizational change. Schools need to foster a values based approach to social accountability. This may involve personalizing social accountability so it means something to all staff, students and others involved in the school, including the health and community sectors. Strategies to influence the organizational culture of the school involve including an understanding of “social accountability” as part of the staff recruitment process (for example in selection criteria or interview questions). Applicants could be asked to describe their understanding of social accountability and how they feel they could contribute to the aspirations of the school. Furthermore, new staff could be inducted into the values of the medical school, by undertaking an orientation on socially accountable practice.

Faculty/staff need to be given opportunities to reflect and act on their own practices; to “walk the talk”. Faculty development could incorporate practical projects on how to engage with underserved communities. Staff in a biomedical or laboratory research role may feel alienated from social accountability. These staff could be given opportunities to learn how social accountability applies to their work. For example, they could develop professional activities that link them with the social accountability agenda of the school. These could include projects with high schools, communities, and research focused on the priority health needs of the communities. However, schools need to appreciate and accept that not all staff will or can have socially accountable practice at the forefront of their work.

## The bedrock: Shared values and aspirations congruent with social accountability

The bedrock of social accountability, that is, the foundation for all building blocks, is shared values and aspirations congruent with social accountability. These values and aspirations are both a philosophical understanding for innovation and a practical application at the health systems and education levels. The values are held by people: the community; leaders; students; faculty and health professionals. These actors or stakeholders aspire to apply these values, often in defiance of ‘mainstream’ health and education systems, by “walking the talk” of social accountability in their everyday work and life.

## Conclusions

The nature of development of social accountability is contextually dependent, politically, historically, socially, spiritually, and economically influenced. Some schools did not term their practice as such until legitimized in this movement for socially accountable medical education. Consequently, development of practice and theory should be seen as iterative rather than prescriptive. Through the process of critical analysis to identify the most important key influences of socially accountable medical education, the building blocks become clear. These are: environmental, including workforce; funding and partnerships; internal to the school, including leadership and community engagement, and about the values of people. There are also examples given in this paper of the actions that medical schools might take to move towards social accountability. The actions are very different depending on the context but if actions are thoughtfully applied and consistent with the building blocks then this might support further movement towards socially accountable medical education.

## Additional file

Additional file 1: Examples of Interview guides. Examples of interview guides for faculty/staff, student, health professional and community representative interviews. (DOCX 18 kb)

## Abbreviations

ADZU SOM: Ateneo de Zamboanga University School of Medicine
FUSOM: Flinders University School of Medicine
GCSA: Global Consensus for Social Accountability
HREC: Human Research Ethics Committees
JCU: James Cook University
JCUSOM: James Cook University School of Medicine
LGU: Local Government Unit
THEnet: Training for Health Equity Network
UPMSHS: University of the Philippines Manila, School of Health Sciences
WHO: The World Health Organization

## Footnotes

1 “Aspiring towards social accountability” is used for schools with an explicit social accountability mandate as social accountability is recognized to be an aspirational ideal.

2 Flinders University, Australia; James Cook University, Australia; Ateneo de Zamboanga University, The Philippines; University of the Philippines, Manila, School of Health Sciences, The Philippines.

3 “A shared understanding of social accountability, in the context of health professional education, was a key inclusion criterion for the cases. If schools and their staff/faculty did not have a shared understanding of social accountability the design flaw would have been “but what is social accountability?” The term could have meant anything and been interpreted in divergent ways. By being members of THEnet schools, the institution had declared that they were aspiring to be socially accountable. Data collection was not distracted by philosophical discussions on what was social accountability and was the school aspiring to be socially accountable” [3] [p.3].
