# Supplementary material for: Building blocks for social accountability: a conceptual framework to guide medical schools
Source: BMC Med Educ. 2016 Aug 26;16(1):227. doi: 10.1186/s12909-016-0741-y (PMC5002162; doi:10.1186/s12909-016-0741-y)
Supplement: Additional file 1: — Examples of Interview guides. Examples of interview guides for faculty/staff, student, health professional and community representative interviews. (DOCX 18 kb) [file 12909_2016_741_MOESM1_ESM.docx]

Example of Semi-structured interview guides

School of Medicine Staff and Students – JCUSOM

| **Question** | **Probes** |
| --- | --- |
| **Introduction**  Could you tell me a bit about yourself and your role at JCUSOM?  *Or*  Could you tell me about bit about yourself, what year are you in medicine? | (may lead onto the School’s story)  What other roles have you had outside of the course, in Sante or RHINO [student clubs]? (student) |
| **Social Accountability**  What’s your understanding of socially accountability in medical education?  What is your understanding of socially accountable medical education in JCUSOM? | Socially accountable medical schools hold themselves responsible for producing outcomes aligned with health workforce, priority health, and health system needs of their communities.  How do you respond to this?  What has influenced socially accountable medical education within the JCUSOM?  What has influenced socially accountable medical education from outside of JCUSOM?  What are the factors that could JCUSOM further progress socially accountable medical education at JCUSOM?  What are some of the barriers? |
| **The School**  Tell me the JCUSOM story?  What do you think are the key broad contextual factors that influence/influenced SA at the School? | Are you aware of the factors that influenced establishment of JCUSOM?/ Why was it established and how?  Who were some of the key people involved in establishing/reorientating the school?  If no, is there anyone else who was around the time of JCUSOM who I should speak with? |
| **Workforce**  How do you perceive the current local medical workforce situation?  What impact has the local medical workforce situation had, in your view, on the JCUSOM?  What about at the national level? | How does this compare with the state/region/nation?  Are there any documents available that I could take away or refer to in terms of the medical workforce? |
| **Health System**  How does the JCUSOM work with the health system?  Has the health system influenced the JCUSOM?  If so, in what ways? For example on the School’s orientation or teaching?  What about at other levels of the health system? | Who does the JCUSOM work with (levels) in the health system?  Do you have a formal agreement?  Who initiates the work?  Could you give me an example of how the JCUSOM works with the Health Sector?  How has this relationship changed over time?  How could this partnership/work be advanced?  What are the challenges?  Are there any documents available that I could take away or refer to? |
| **Community**  What is your understanding of the JCUSOM’s relationship with the community?  Who do you see as the community?  How much influence do you feel the community has on JCUSOM?  How do communities get influence? | Who do you see as JCUSOM’s community?  Who initiates partnerships?  Could you give me an example of a partnership with the community?  Why do you partner with the community? /What are the motivations for partnering with the community?  Do you have a formal agreement or advisory committee or other formal structures?  What do you see as the benefits of these partnerships?  How could they be advanced?  What are the challenges?  How has this relationship/partnership changed over time? |
| Who else could follow up with/interview? |  |
| Are there any documents available that I could take away or refer to? |  |

**Health Sector Questions – JCUSOM**

| **Question** | **Probes** |
| --- | --- |
| **Introduction**  Could you tell me a bit about yourself and your involvement with JCUSOM? | *(may lead onto the School’s story)* |
| **Social Accountability**  What’s your understanding of socially accountability in medical education?  What is your understanding of socially accountable medical education in JCUSOM? | *Socially accountable medical schools hold themselves responsible for producing outcomes aligned with health workforce, priority health, and health system needs of their communities.*  How do you respond to this?  What has influenced socially accountable medical education within the JCUSOM?  What has influenced socially accountable medical education from outside of JCUSOM?  What are the factors that could JCUSOM further progress socially accountable medical education at JCUSOM?  What are some of the barriers? |
| **The School**  Tell me the school’s story?  What do you think are the key broad contextual factors that influence/influenced SA at the School? | Are you aware of the factors that influenced establishment of JCUSOM?/ Why was it established and how?  Who were some of the key people involved in establishing/reorientation of the JCUSOM?  If no, is there anyone else who was around the time of JCUSOM/changes at JCUSOM who I should speak with? |
| **Health Sector**  What is your organisation’s relationship with JCUSOM? | Do you have a formal agreement?  Who initiates the work?  Could you give me an example of how you work with the JCUSOM?  How could this partnership/work be advanced?  What are the challenges?  Has the partnership changed over time?  Are there any documents available that I could take away or refer to? |
| What was your organisation’s involvement at the establishment of JCUSOM?/or the reorientation?  (*If not organisation/the health sector?)* | Are you aware of the factors that influenced establishment of JCUSOM?  If no, is there anyone else who was around the time of JCUSOM  Are there any documents available that I could take away or refer to? |
| How has your organisation influenced the JCUSOM?  If so, in what ways? For example on the School’s orientation or teaching?  What about at other levels of the health system | How could you or would you like to influence JCUSOM policies? |
| **Workforce**  How do you perceive the current local medical workforce situation?  What impact has the local medical workforce situation had, in your view, on the JCUSOM?  What about at the national level? | How does this compare with the state/region/nation?  How has JCUSOM worked with the health sector? (in terms of workforce)  What is the impact of student placements on the health system? (positive/negative)  Are there any documents available that I could take away or refer to in terms of the medical workforce? |
| **Community**  What is your understanding of the JCUSOM’s relationship with the community?  How much influence do you feel the community has on JCUSOM?  How do communities get influence? | How has the JCUSOM had to adapt for community need  What do you see as the benefits of these partnerships?  How could they be advanced?  What are the challenges?  How has this relationship/partnership changed over time?  Are there any documents available that I could take away or refer to? |
| Who else could follow up with/interview? |  |
| Are there any documents available that I could take away or refer to? |  |

**Community Questions - FUSOM**

| **Question** | **Probes** |
| --- | --- |
| **Introduction** | Could you tell me a bit about yourself and your involvement with FU SOM? |
| **Social Accountability**  What’s your understanding of socially accountability in medical education?  What is your understanding of socially accountable medical education in FUSOM? | *Socially accountable medical schools hold themselves responsible for producing outcomes aligned with health workforce, priority health, and health system needs of their communities.*  How do you respond to this?  What has influenced socially accountable medical education within the FUSOM?  What has influenced socially accountable medical education from outside of FUSOM?  What are the factors that could FUSOM further progress socially accountable medical education at FUSOM?  What are some of the barriers? |
| **The School**  Tell me the FUSOM story?  What do you think are the key broad contextual factors that influence/influenced SA at the School? | Are you aware of the factors that influenced establishment of FU SOM?/ Why was it established and how?  Who were some of the key people involved in establishing/reorientation of the FU SOM?  If no, is there anyone else who was around the time of FU SOM/changes at FU SOM who I should speak with? |
| **Community**  What is your understanding of the FU SOM’s relationship with the community? | What is your understanding of the FU SOM’s community or reference population? / Who do you see as FU SOM’s community?  What do you see as your role with the medical school?/  What do you do with the medical school?/How do you partner with the medical school?  Why do you partner/or are involved with the medical school?  /What are the motivations for partnering with/being involved with the medical school?  Do you have a formal agreement or advisory committee or other formal structures?  Who initiates partnerships?  Could you give me an example of a partnership with the community?  What do you see as the benefits of these partnerships?  How could they be advanced?  What are the challenges?  How has this relationship/partnership changed over time?  Are there any documents available that I could take away or refer to? |
| How much influence do you feel the community has on FUSOM?  How has your organisation influenced the FUSOM?  If so, in what ways? For example on the School’s orientation or teaching? | How could you or would you like to influence FUSOM policies?  How do communities get influence?  How has the FU SOM had to adapt for community need?  What about at other sectors of the community? |
| What was the community’s/your organisation’s /your involvement at the establishment of FUSOM?/or the reorientation? | Are you aware of the factors that influenced establishment of FUSOM?  If no, is there anyone else who was around the time of FUSOM  Are there any documents available that I could take away or refer to? |
| **Health Sector**  What is your understanding of the FU SOM’s relationship with the Health Sector? | Could you give me an example how the FU SOM works with the health sector?  How could this partnership/work be advanced?  What are the challenges?  Has the partnership changed over time?  Are there any documents available that I could take away or refer to? |
| **Workforce**  How do you perceive the current local medical workforce situation?  What impact has the local medical workforce situation had, in your view, on the FU SOM?  What about at the national level? | How does this compare with the state/region/nation?  Could you give me an example of how has FU SOM worked with the health sector? (in terms of workforce)  What is the impact of student placements on the health system? (positive/negative)  Are there any documents available that I could take away or refer to in terms of the medical workforce? |
| Who else could follow up with/interview? |  |
| Are there any documents available that I could take away or refer to? |  |
